# Which Surgical Operations Should be Performed in District Hospitals in East, Central and Southern Africa? Results of a Survey of Regional Clinicians

**DOI:** 10.1007/s00268-020-05793-8

**Published:** 2020-09-30

**Authors:** Zineb Bentounsi, Chris Lavy, Chiara Pittalis, Morgane Clarke, Jean Rizk, Grace Le, Ruairi Brugha, Eric Borgstein, Jakub Gajewski

**Affiliations:** 1 Nuffield Department of Orthopaedics, Rheumatology and Musculoskeletal Sciences, Botnar Research Centre, University of Oxford, Old Road, Oxford, OX3 7LD UK; 2 Royal College of Surgeons in Ireland, Beaux Lane House, Dublin, 2 Ireland; 3 Department of Surgery, Queen Elizabeth Central Hospital, Chipatala Avenue, PO Box 95, Blantyre, Malawi

## Abstract

**Background:**

In East, Central and Southern Africa (ECSA), district hospitals (DH) are the main source of surgical care for 80% of the population. DHs in Africa must provide basic life-saving procedures, but the extent to which they can offer other general and emergency surgery is debated. Our paper contributes to this debate through analysis and discussion of regional surgical care providers' perspectives.

**Methods:**

We conducted a survey at the College of Surgeons of East, Central and Southern Africa Conference in Kigali in December 2018. The survey presented the participants with 59 surgical and anaesthesia procedures and asked them if they thought the procedure should be done in a district level hospital in their region. We then measured the level of positive agreement (LPA) for each procedure and conducted sub-analysis by cadre and level of experience.

**Results:**

We had 100 respondents of which 94 were from ECSA. Eighteen procedures had an LPA of 80% or above, among which appendicectomy (98%), caesarean section (97%) and spinal anaesthesia (97%). Twenty-one procedures had an LPA between 31 and 79%. The surgical procedures that fell in this category were a mix of obstetrics, general surgery and orthopaedics. Twenty procedures had an LPA below 30% among which paediatric anaesthesia and surgery.

**Conclusion:**

Our study offers the perspectives of almost 100 surgical care providers from ECSA on which surgical and anaesthesia procedures should be provided in district hospitals. This might help in planning surgical care training and delivery in these hospitals.

**Electronic supplementary material:**

The online version of this article (10.1007/s00268-020-05793-8) contains supplementary material, which is available to authorized users.

## Introduction

In sub-Saharan Africa (SSA), district hospitals (DH) or Level 1 hospitals according to The World Health Organisation (WHO) are the first level of hospital that provide in-patient surgery and anaesthesia [1]. DHs are of crucial importance in SSA because for 80% of the population they are the main source of hospital care [2]. In general, they cater for essential and emergency surgical care for populations of 100,000–500,000.

There is a wide disparity in resources available at DHs in the East, Central and Southern Africa (ECSA) region [3]. Very few district hospitals in the region have a formally trained surgeon [4], in the majority surgical care is provided by either non-specialist doctors or non-physician clinical officers, and anaesthesia is mainly performed by clinical officers or anaesthetic nurses [5].

DHs in Africa must provide basic life-saving procedures, but the extent to which they can offer other general and emergency surgery is debated. In 1992, a WHO publication [6] outlined a detailed list of surgical procedures recommended for DHs (see box 1). There have also been similar lists suggested in the 2018 Disease Control Priorities Essential Surgery volume (2). The above lists are useful, but they are general in nature and not specific to the ECSA region.

**Box 1:** Operations that should be available at the district hospital (in the hospital in rural and urban districts: report of a WHO Study Group on the Functions of Hospitals at the first referral 1992) [6]

General surgery	Obstetrics and gynaecology	Orthopaedics and trauma
Biopsies	Caesarean section	Amputations
Circumcision	Craniotomy	Plaster techniques
Extraction of teeth	Delivery	Traction (skull, limbs)
Laparotomy	Dilatation and curettage	Management of:
Tracheostomy	Episiotomy	Bone fractures
Bladder puncture	Evacuation of uterine cavity	Burn wounds
Colostomy	Insertion and removal of IUD	Dislocation of joints
Feeding gastrostomy	Management of:	Head injuries
Management of:	Cord prolapse	Spinal injuries (without cord damage)
Abdominal wall hernias	Ectopic pregnancy	
Anal fissures and fistulae	Ruptured uterus	
Fluid and electrolyte balance	Ruptured cervix	
Intestinal obstruction	Ruptured perineum	
Intussusception	Ruptured vagina	
Perforated intestine, ulcers	Sterilization, female	
Catheterization	Symphysiotomy	
Control of epistaxis	Version and extraction	
Hydrocelectomy		
Removal of foreign body		
Transport of severely injured patients		
Cholecystectomy		
Debridement and care of wounds		
Incision and drainage of abscesses		
Sterilization, male		
Anaesthesia:	Care of airways	Conduction anaesthesia
Anaesthesia for emergency cases	Intubation and management of its complications	Postoperative recovery care
Induction and maintenance of general anaesthesia		

The Lancet Commission on Global Surgery [7] also questions which surgical procedures should be performed at a district level and recommends that the list of suggested generic DH surgical procedures in the Commission Report (based on the WHO 1992 publication) is updated and adapted to country/region specific contexts [7]. In the ECSA region, one of the contexts that has changed for the better is the increased numbers of surgeons trained since the establishment of the College of Surgeons of East Central and Southern Africa (COSECSA) [8] in 1999. Our study contributes to understanding of which surgical procedures should be available at district level in ECSA by collecting and analysing the views of operating theatre personnel working in the region.

## Methodology

### Survey design

A list of 59 surgical and anaesthesia procedures was developed based on the Surgeons OverSeas personnel, infrastructure, procedures, equipment and supplies (PIPES) tool [9]. This list of procedures was modified by removing some of the simpler procedures such as ‘suturing’, and ‘drainage of abscesses’ for which there would be no dispute and adding some more controversial complex procedures that have been done at some district hospitals in the region. The aim was not to have an unwieldy comprehensive list of every possible procedure, but a representative range that went from simple to complex.

Laparotomy is on the PIPES list but is not clearly defined. We decided to leave it in the list, but we added specific procedures that would need doing if found at laparotomy, e.g. bowel resection, anastomosis, stoma, excision of mass, splenectomy, trauma.

These procedures were listed on a printed survey, and participants were asked for a yes/no response on whether each procedure should be performed in DHs in their country. Participants responded anonymously, but gave demographic information on their job title, qualification, country of work and experience of surgery at district hospitals in ECSA.

### Survey administration and ethics

With advance approval obtained from the COSECSA Education and Scientific Committee, the survey was distributed in Kigali in December 2018 at the annual COSECSA conference. The survey was announced in the plenary session at the conference and then, distributed by 3 researchers to any participant who declared an interest in ECSA DH surgery and wished to contribute. This study was covered by the Research Ethics Committee of the Royal College of Surgeons in Ireland under approval No. REC 1417.

### Subgroup definitions

For analysis, we classified as “anaesthetist” both physicians and non-physician anaesthesia providers and we conducted a sub-analysis of anaesthetists’ opinion. We classified respondents as “experienced” if they had worked in DHs in ECSA, and as “non-experienced”, if they had not. We statistically tested the difference in opinion between the two groups with the Wilcoxon test. All analysis was conducted in R software environment version 3.5.2 [10], and the level of confidence was set at 95%.

### Validation of the responses

To validate the survey answers for their internal consistency (i.e. whether respondents completed the survey in a consistent manner), we used Cronbach's alpha [11]. To further test the internal consistency of the responses, we checked the correlation between responses in relation to complex and less complex procedures of the same surgical specialty. For example, if a respondent agrees that a relatively complex gynaecological procedure, such as hysterectomy, should be done at DHs, then they would be expected to agree that a less complex procedure, such as caesarean section, should also be done at DHs. Thus, seven pairs of procedures (complex and less complex) were chosen (Table 1).

**Table 1:** Paired procedures of the same surgical specialty. The last column is the percentage of respondents who answered ‘yes’ to the complex procedure and ‘no’ to the less complex one

Specialty	A—Complex procedure	B—Less complex procedure in same specialty	Yes for A and No for B (%)
Gynaecology	Hysterectomy	Caesarean section	0
General surgery	Paediatric hernia repair	Elective hernia repair	0
Orthopaedics	Treatment of open or compound fracture	Traction closed fracture	0
Ortho/General	Amputation above knee	Amputation below knee	0
Paediatric surgery	Gastroschisis surgery	Paediatric hernia repair	1
Chest/airway	Cricothyroidotomy	Chest drain insertion	1
Plastics	Cleft lip repair	Skin grafting	4

### Level of positive agreement

We measured the Level of Positive Agreement (LPA) for each procedure. Thus, 100% LPA for a procedure meant that all participants agreed the procedure should be done at a DH, while 0% LPA meant that no participant felt the procedure should be done at a DH.

## Results

### Survey participants

**Fig. 1:**
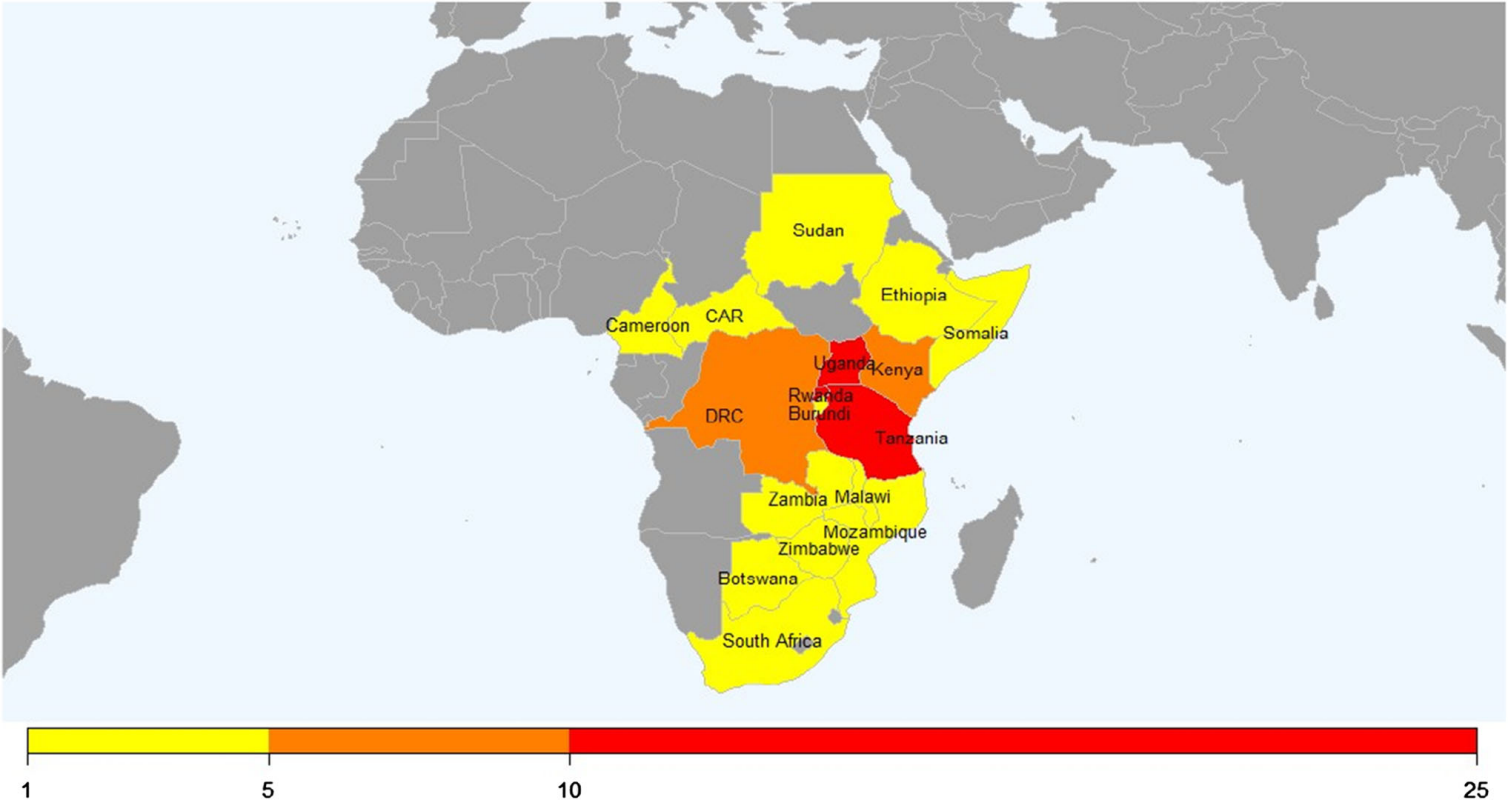
Geographical distribution of African respondents

The survey was completed by 100 respondents from 21 countries, of whom 94 were from Africa (see map in Fig. 1); with the majority coming from Rwanda, Uganda and Tanzania. Six respondents were based outside Africa and came from the USA, Ireland, Scandinavia and New Zealand, 79 respondents were surgeons (see Table 2).

**Table 2:** Characteristics of respondents

Characteristics	Number of respondents
Surgeons	79
Surgical specialty	
General surgery	14
Neurosurgery	04
Paediatric surgery	06
Orthopaedics	09
Obstetrics and Gynaecology	01
Urology	05
ENT	01
Plastics	01
Unspecified	38
Qualification level	
In surgical training	09
Post training	67
Unknown	03
Non-surgeons	19
Profession	
Medical Officers	09
Medical Student	01
Nurse	01
Medically Qualified Anaesthetists	02
Non Physician Anaesthetists	04
Other Medical Doctors	02

### Validation of Responses

The Cronbach's alpha for our survey is 0.91, which shows a high level of consistency. For the first 4 of the 7 pairs of complex/less complex procedures (shown in Table 1), all the respondents’ answers were consistent, i.e. none suggested that a complex procedure should be done in DHs while a less complex one should not. For the next 2 pairs, there was one inconsistent respondent, and, for the last pair, *cleft lip repair/skin grafting*, 4 respondents suggested that *skin grafting* should not be done, while *cleft lip repair* should*.*

**Fig. 2:**
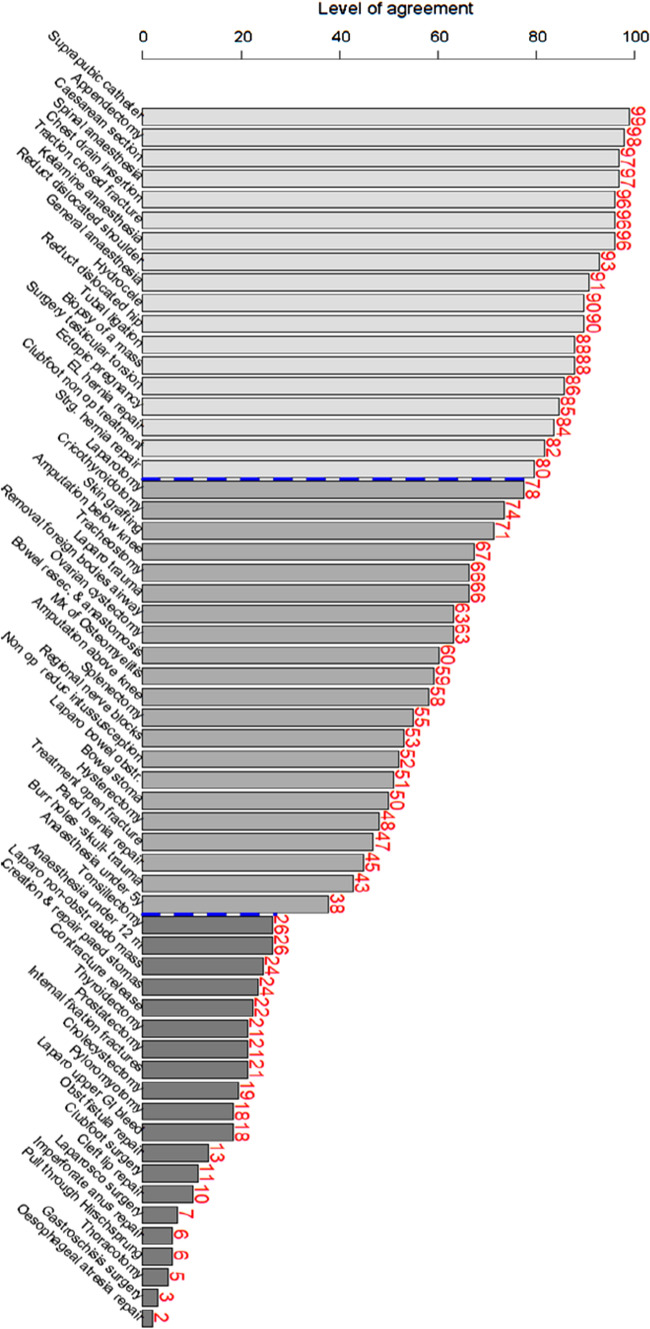
Graphic representation of Level of Positive Agreement

The level of agreement for each procedure is shown in Fig. 2 (and Supplemental Table 1). On the left of Fig. 2 are the procedures where there is high agreement that they should be done at DHs; on the right are the procedures with least agreement. We arbitrarily identified three broad groups of procedures. Group 1: those procedures that a large majority think should definitely be done at DHs. Group 2: those where there was debate as to whether they should be done, and Group 3: those that a large majority thought should not be done. The three groups are identified by a different colour in Fig. 2, Group 1 is on the left, Group 2 in the middle and Group 3 on the right. There are dotted lines between the groups. Please note that these groups and the thresholds are arbitrary and for discussion only.

#### Group 1

LPA of 80% or above, consisting of 18 procedures with broad consensus of 80% or more of participants that they should be done at DHs. The surgical procedure with the highest LPA was suprapubic catheterisation (99%), followed by appendicectomy (98%) and caesarean section (97%). The anaesthesia procedure with the highest LPA was spinal anaesthesia (97%). Ketamine anaesthesia reached 96% positive agreement and general anaesthesia for adults 91% (Fig. 2).

#### Group 2

LPA of 31–79%, consisting of 21 procedures. There were two anaesthesia procedures in this category: regional nerve blocks (LPA = 53%) and anaesthesia administered to children under 5 years old (LPA = 38%). The surgical procedures that fell in this category were a mix of obstetrics, general surgery and orthopaedics. It is of note that all types of laparotomy fell into this category. Large bowel obstruction (51%), stoma formation (50%) and hysterectomy (48%) were in the middle of the group with almost half of respondents recommending that they were done, and the other half disagreeing.

#### Group 3

LPA of below 30%, consisting of 20 procedures. Administering anaesthesia to children under 12 months and tonsillectomy had an LPA of 26%. The three procedures with the lowest LPA were thoracotomy (5%), gastroschisis surgery (3%) and oesophageal atresia repair (2%).

### Subgroup Differences

The Wilcoxon test showed no significant difference in terms of LPA (*p*-value = 0.3) between those we classified as “experienced” in DH surgery (84 respondents) and those “non-experienced” (14 respondents). No significant differences were found for all the other demographics characteristics (country of work, profession, qualification) of study participants in relation to their responses.

All 6 anaesthetists agreed that general, spinal, ketamine and local anaesthesia should be done at a DH. All but one anaesthetist agreed that regional blocks should not be administered at a DH, while 53% of non-anaesthetist respondents think they should be offered. Similarly, all but one anaesthetist agreed that paediatric anaesthesia should not be administered at DHs.

## Discussion

We found a high level of general agreement over the procedures that should be done at a DH (Group 1), and on those that should not be done (Group 3). There remains discussion and disagreement on the Group 2 procedures. It is perhaps unsurprising that the level of positive agreement (Fig. 2) largely reflects the degree of complexity of the procedure, which may reflect respondents’ knowledge about the training and confidence of the DH staff in their home countries.

We found that the areas of agreement and disagreement were similar between those who had worked in district hospitals (“experienced”) and those who had not (“non-experienced”). This perhaps reflects the fact that concerned clinicians in a region do not need to actually work in the DHs to understand the situations in DHs and to recognise key issues. Indeed, clinicians who do not themselves work in DHs but take surgical referrals from DHs should be expected to have valid opinions on what DHs are capable of doing.

All common adult intra-abdominal surgery (that was recommended in WHO 1992), including bowel obstruction, bowel resection/anastomosis and hysterectomy, was in Group 2. The pathology requiring these operations is common and often life threatening; if surgery is not available, then there are significant risks to the patients in transfer, especially when the journey to the referral hospital is difficult. As an outsider to the region, one might think that these cases should definitely be available in DHs; however, it is possible that current DH staff have insufficient training in abdominal surgery, or they are more risk averse in today’s society, and also that road transfer to a referral centre is easier now than it was 30 years ago.

Surgical camps may have influenced some of our respondents' answers. These camps periodically bring manpower and resources that are not normally present at DHs but can massively increase elective surgical output. Plastic surgery teams are one of the common visiting groups; thus, the respondents who felt that cleft lips could be done at DHs, but not skin grafting, may have worked in a DH where elective cleft lips were done regularly by a visiting plastic surgical team, but felt that skin grafting for injuries in the absence of a plastic surgeon was too difficult or even impossible without the necessary equipment.

The risks of general anaesthesia in children is probably the reason that so many respondents thought this should not be done in DHs. Surgeons felt that regional blocks were appropriated in DHs, while anaesthetists disagreed. The reason for this difference of opinion is unclear; perhaps, it is because anaesthetists have a better understanding of the difficulty of effective blocks and felt safer with a more reliable standard general anaesthetic which they did regularly and knew well. In our previous work, regional blocks were found to be commonly done in Malawi and Zambia but not in Tanzania [12].

**Fig. 3:**
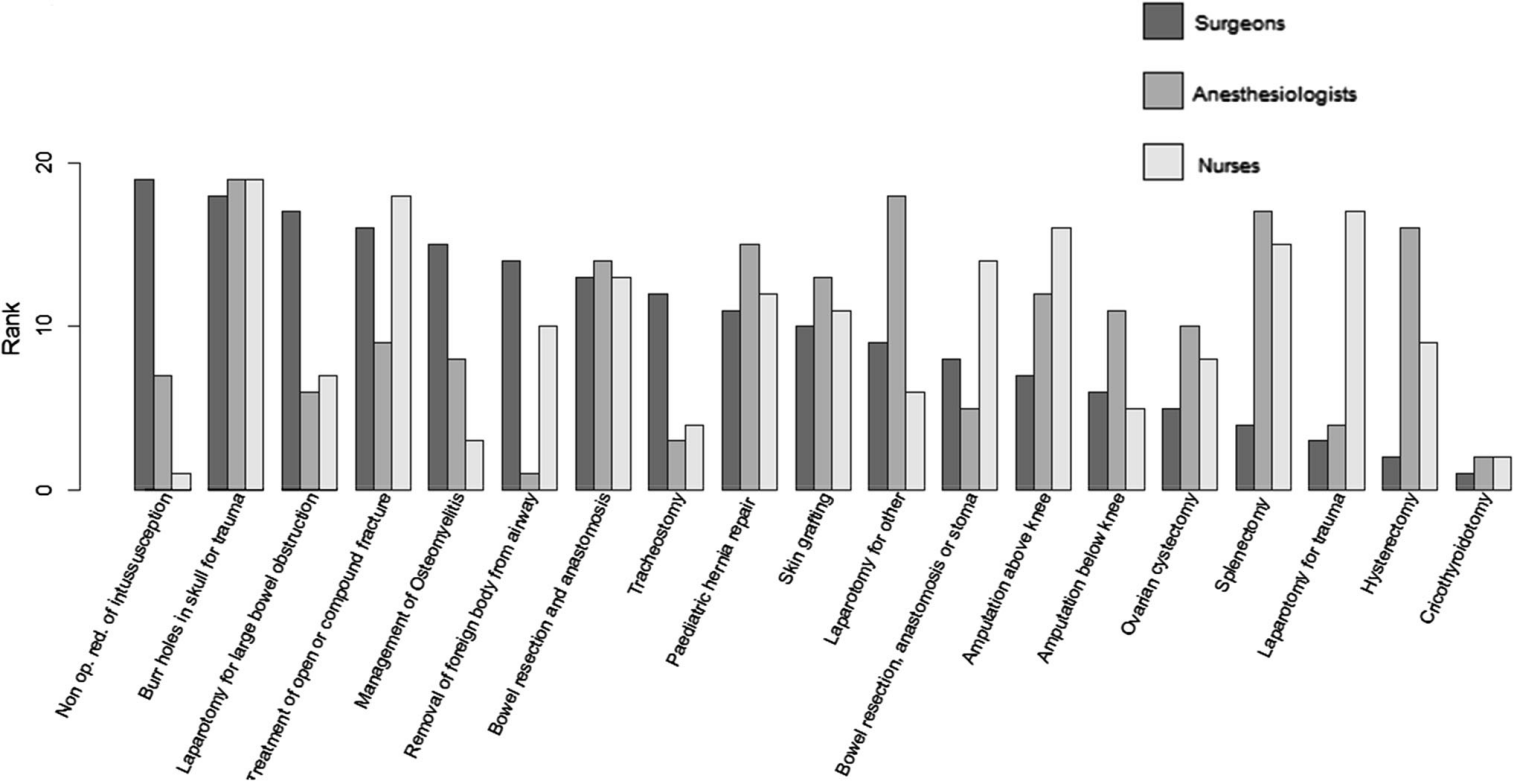
Ranking of the 19 surgical procedures in Group 2 for appropriateness in a DH by different cadres of surgical workforce in Zambia (note that rank 1 means most suitable and rank 19 least suitable)

As this study was part of an implementation research project, we shared the results with a group of 9 surgeons, 9 anaesthetists and 10 theatre nurses in Zambia, all of whom worked regularly in DHs. All agreed that the procedures in Group 1 should be done and those in Group 3 should not be done in DHs. Group 2 procedures, however, were controversial. In order to compare the cadres’ viewpoints, we randomly assorted the 19 surgical procedures in Group 2 onto moveable cards, then asked each cadre group independently to gain consensus and line up the cards in order of increasing suitability for a district hospital. Figure 3 shows the rankings for each procedure and cadre.

It is particularly poignant to see that surgeons were generally comfortable to take on splenectomy and laparotomy for trauma, while the more cautious nurses and anaesthetists disagreed. Perhaps, they had seen too often the results of overoptimistic surgeons and long operations or excessive bleeding. The different cadres may also have judged a procedure on the degree of difficulty of their part in the procedure rather than the overall procedure itself. These differences are an important reminder that different team members have different opinions. Surgery requires teamwork, and research has shown that when all members of the team are part of decision making, patient safety improves [13].

Further research is needed to determine management outcomes for patients with conditions requiring procedures that respondents said should not be done in DHs (Group 3). Some might be referred to Level 2 hospitals and receive treatment, as shown by our previous work in Malawi where 80% of cases referred to the central hospital came from DHs [14]. But some might not get any treatment, indeed The African Surgical Outcomes Study (ASOS) estimates that only 4% of needed surgery in the continent actually ever gets done [15].

## Strengths and limitations

The strength of this study is that it is the first study that we have seen that looks at the opinion of almost 100 local involved clinicians on what surgery should be done at an ECSA DH. It also provides perspectives of different cadres involved in surgical care (i.e. anaesthetists, nurses and non-physician clinicians) from 15 countries across the region. However, this study has limitations. First, our survey at the COSECSA conference involved only self-selected individuals. Second, our study only looked at opinions on what surgical procedures individual clinicians felt should be offered at a DH; it did not look at population needs or assess the burden of disease.

Third, there could have been some confusion in terms. The survey presented several categories of laparotomy, for example, and the term ‘management of open fractures’ could have been interpreted by some as ‘initial management’ (such as debridement and wound management), and by others as ‘definitive’ (involving frames, internal nailing and dealing with complex flaps and non-unions). Similarly, laparotomy and open fracture treatment were chosen as bellwether conditions in the Lancet Commission on Global Surgery, but the procedure required to treat them was not clearly defined [7]. This lack of clarity has led other authors to consider moving beyond Bellwether procedures as a metric and using baskets of procedures instead [16]. For future surveys and guidance, a clearer definition of procedures would be helpful.

Another limitation of our study is that we did not ask for reasons why a procedure should or should not be available in a DH. This would have made the questionnaire longer but could be considered in further surveys.

## Conclusion

This study is a comment on the need and practicality of surgery in district hospitals in sub-Saharan Africa and is not a blueprint for what should actually be offered. Nevertheless, our study provides the opinions of almost 100 practicing clinicians in the surgical workforce. It should be useful for health planners and funders in designing surgical systems, and to Ministry of Health personnel designing National Surgical Obstetric and Anaesthesia Plans [17].

Our sub-analysis by profession and the discussion of our results in cadre groups have highlighted a difference in perception between members of the surgical team as to the appropriateness of some procedures for DHs. We suggest this is an area for further discussion within surgical teams, and for national surgical training programmes to consider the whole surgical team when designing training.

We noted earlier the disparity in healthcare resources across the ECSA countries; it may be appropriate for COSECSA to develop a ladder of surgical procedures moving from simple to complex. The ECSA countries are increasingly including surgery in their health planning, and they could use such a ladder and define agreed cut off points for their countries DHs or for particular hospitals in order to clarify what procedures are appropriate and safe to be performed in DHs or in particular DHs. If this is done, then a team training programme could be developed that follows this ladder of complexity, and this could be taught by visiting specialists to DH staff and used as a mentoring tool on further visits.

## Electronic supplementary material

Below is the link to the electronic supplementary material.

Supplementary file1 (DOCX 21 kb)
