# Supplementary material for: Which Surgical Operations Should be Performed in District Hospitals in East, Central and Southern Africa? Results of a Survey of Regional Clinicians
Source: World J Surg. 2020 Sep 30;45(2):369–77. doi: 10.1007/s00268-020-05793-8 (PMC7773610; doi:10.1007/s00268-020-05793-8)
Supplement: Supplementary file 1 — Supplementary file1 (DOCX 21 kb) [file 268_2020_5793_MOESM1_ESM.docx]

Supplementary Table 1: Level of Positive Agreement per Procedure

| **Surgical and Anaesthesia Procedures** | **Number of positive votes** | **Level of Positive Agreement** |
| --- | --- | --- |
| Suprapubic catheter | 97 | 99.0% |
| Appendectomy | 96 | 98.0% |
| Caesarean section | 95 | 96.9% |
| Spinal anaesthesia | 95 | 96.9% |
| Chest drain insertion | 94 | 95.9% |
| Traction closed fracture | 94 | 95.9% |
| Ketamine anaesthesia | 94 | 95.9% |
| Reduct. dislocated shoulder | 91 | 92.9% |
| General anaesthesia | 89 | 90.8% |
| Hydrocele | 88 | 89.8% |
| Reduct. dislocated hip | 88 | 89.8% |
| Tubal ligation | 86 | 87.8% |
| Biopsy of a mass | 86 | 87.8% |
| Surgery testicular torsion | 84 | 85.7% |
| Ectopic pregnancy | 83 | 84.7% |
| Elective hernia repair | 82 | 83.7% |
| Clubfoot non op treatment | 80 | 81.6% |
| Strg. hernia repair | 78 | 79.6% |
| Laparotomy | 76 | 77.6% |
| Cricothyroidotomy | 72 | 73.5% |
| Skin grafting | 70 | 71.4% |
| Amputation below knee | 66 | 67.3% |
| Tracheostomy | 65 | 66.3% |
| Laparo. trauma | 65 | 66.3% |
| Removal foreign bodies airway | 62 | 63.3% |
| Ovarian cystectomy | 62 | 63.3% |
| Bowel resec. & anastomosis | 59 | 60.2% |
| Mx of Osteomyelitis | 58 | 59.2% |
| Amputation above knee | 57 | 58.2% |
| Splenectomy | 54 | 55.1% |
| Regional nerve blocks | 52 | 53.1% |
| Non op. reduc. intussusception | 51 | 52.0% |
| Laparo. bowel obstr. | 50 | 51.0% |
| Bowel stoma | 49 | 50.0% |
| Hysterectomy | 47 | 48.0% |
| Treatment open fracture | 46 | 46.9% |
| Paed. hernia repair | 44 | 44.9% |
| Burr holes -skull- trauma | 42 | 42.9% |
| Anaesthesia under 5y | 37 | 37.8% |
| Tonsillectomy | 26 | 26.5% |
| Anaesthesia under 12 m | 26 | 26.5% |
| Laparo. non-obstr. Abdo. mass | 24 | 24.5% |
| Creation & repair paed. stomas | 23 | 23.5% |
| Contracture release | 22 | 22.4% |
| Thyroidectomy | 21 | 21.4% |
| Prostatectomy | 21 | 21.4% |
| Internal fixation fractures | 21 | 21.4% |
| Cholecystectomy | 19 | 19.4% |
| Pyloromyotomy | 18 | 18.4% |
| Laparo. upper GI bleed | 18 | 18.4% |
| Obst. fistula repair | 13 | 13.3% |
| Clubfoot surgery | 11 | 11.2% |
| Cleft lip repair | 10 | 10.2% |
| Laparoscopic surgery | 7 | 7.1% |
| Imperforate anus repair | 6 | 6.1% |
| Pull through Hirschsprung | 6 | 6.1% |
| Thoracotomy | 5 | 5.1% |
| Gastroschisis surgery | 3 | 3.1% |
| Oesophageal atresia repair | 2 | 2.0% |
